# Suprachoroidal effusion with angle-closure glaucoma in a patient with active systemic lupus erythematosus: a case report

**DOI:** 10.1186/s12886-026-04971-x

**Published:** 2026-05-30

**Authors:** Xi Guo, Yan Li, Juan Wang, Yan Zhang, Pengcheng Wu

**Affiliations:** 1 Department of Ophthalmology, The Second Hospital & Clinical Medical School, Lanzhou University, No. 82 Cuiyingmen, Lanzhou, Gansu Province 730030 China; 2 Gansu Province Clinical Research Center for Ophthalmology, No. 82 Cuiyingmen, Lanzhou, Gansu Province 730030 China; 3 Department of Rheumatology, The Second Hospital & Clinical Medical School, Lanzhou University, Lanzhou, China

**Keywords:** Uveal effusion syndrome, Systemic lupus erythematosus, Angle-closure glaucoma, Hormonotherapy, Choroidal detachment

## Abstract

**Background:**

Systemic lupus erythematosus (SLE) uncommonly presents with ocular manifestations as the initial feature. Uveal effusion syndrome (UES) is typically described in eyes with nanophthalmos and scleral abnormalities. This case is noteworthy for two reasons. First, the patient had normal axial length and no marked diffuse scleral thickening on ultrasound biomicroscopy(UBM), with a massive annular uveal/suprachoroidal effusion resembling a type III UES-like phenotype; however, concurrent active SLE, hypoalbuminaemia and polyserositis suggested a secondary systemic trigger. Second, ocular recovery appeared to accelerate after periocular corticosteroid therapy, despite inadequate response to systemic immunosuppression.

**Case presentation:**

A 38-year-old woman presented with three days of bilateral ocular pain and visual deterioration. Initial examination revealed intraocular pressure of 38.7 mmHg in the right eye and 32.7 mmHg in the left, shallow anterior chambers, and axial length of 21.85 mm bilaterally. Bilateral angle-closure glaucoma was diagnosed, and laser peripheral iridotomy was performed along with topical antiglaucoma agents and corticosteroids. One week later, systemic symptoms including fever and polyarthralgia developed. Laboratory tests showed strongly positive autoimmune markers (ANA: antinuclear antibody, ANSA༚anti-nucleosome antibody, ARPA༚anti-ribosomal P protein antibody), low complement levels (C3༚complement 3, C4༚complement 4). Inflammatory markers were elevated (CRP༚C-reactive protein 84.08 mg/L, ESR༚erythrocyte sedimentation rate 83 mm/h), severe hypoalbuminaemia (27.4 g/L). Imaging revealed pleural, pericardial, and peritoneal effusions. Active SLE with polyserositis and lupus nephritis was diagnosed. Intravenous methylprednisolone, cyclophosphamide, hydroxychloroquine and telitacicept were initiated. However, ultrasonography showed progressive 360-degree annular choroidal detachment with anterior displacement of the lens-iris diaphragm. Bilateral periocular triamcinolone acetonide 20 mg was administered, followed by periocular dexamethasone 5 mg. Choroidal detachment rapidly resolved, anterior chamber depth increased from 1.57 mm to 2.93 mm, and the retina reattached spontaneously. One month later, best-corrected visual acuity improved from 20/500 to 20/50 in both eyes, and intraocular pressure normalised.

**Conclusions:**

This case illustrates that systemic serous leakage in SLE can cause massive suprachoroidal effusion with secondary angle-closure glaucoma, even in eyes with normal axial length. Periocular corticosteroid therapy may serve as a useful adjunct by increasing local ocular drug exposure and promoting fluid resorption.

## Introduction

Systemic lupus erythematosus (SLE) has an incidence of 7 to 29% of the involvement of the posterior segment of the eye. Posterior segment manifestations are most commonly attributed to immune complex-mediated vascular injury, presenting as lupus retinopathy or choroidopathy [1].Uveal effusion syndrome (UES), by contrast, is a rare entity characterised by annular non-rhegmatogenous choroidal detachment, typically occurring in eyes with nanophthalmos or abnormal scleral permeability [2]. We report a case of active SLE presenting with bilateral acute angle-closure glaucoma secondary to massive circumferential suprachoroidal effusion in eyes with normal axial length, posing a significant diagnostic challenge.

## Materials and methods

This study was conducted as a single observational case report at The Second Hospital & Clinical Medical School, Lanzhou University. The patient provided written informed consent before participating in the study.

## Results

### Case report

A 38-year-old woman presented with a three-day history of bilateral ocular pain and sudden visual deterioration. She had no previous ocular disease. Best-corrected visual acuity (BCVA) was 20/500 in both eyes. Intraocular pressure measured 38.7 mmHg in the right eye and 32.7 mmHg in the left. The patient’s axial length was 21.85 mm in both eyes. Slit-lamp examination showed shallow anterior chambers, mid-dilated pupils, and sluggish light reflexes bilaterally.

Bilateral angle-closure glaucoma was diagnosed. The patient underwent laser peripheral iridotomy (PI). Topical antiglaucoma agents and corticosteroids were also administered. Despite treatment, visual acuity deteriorated further. One week later, systemic symptoms including fever and polyarthralgia developed.

Laboratory evaluation revealed that autoimmune markers(ANA, ANSA, ARPA)were strongly positive; complement levels (C3, C4) were significantly decreased; Inflammatory markers CRP was 84.08 mg/L, ESR was 83 mm/h; severe hypoalbuminaemia (27.4 g/L), hyponatremia and hemolytic anemia were also present. Imaging demonstrated pleural, pericardial, and peritoneal effusions. A diagnosis of active SLE with polyserositis and lupus nephritis was established.

**Fig. 1:**
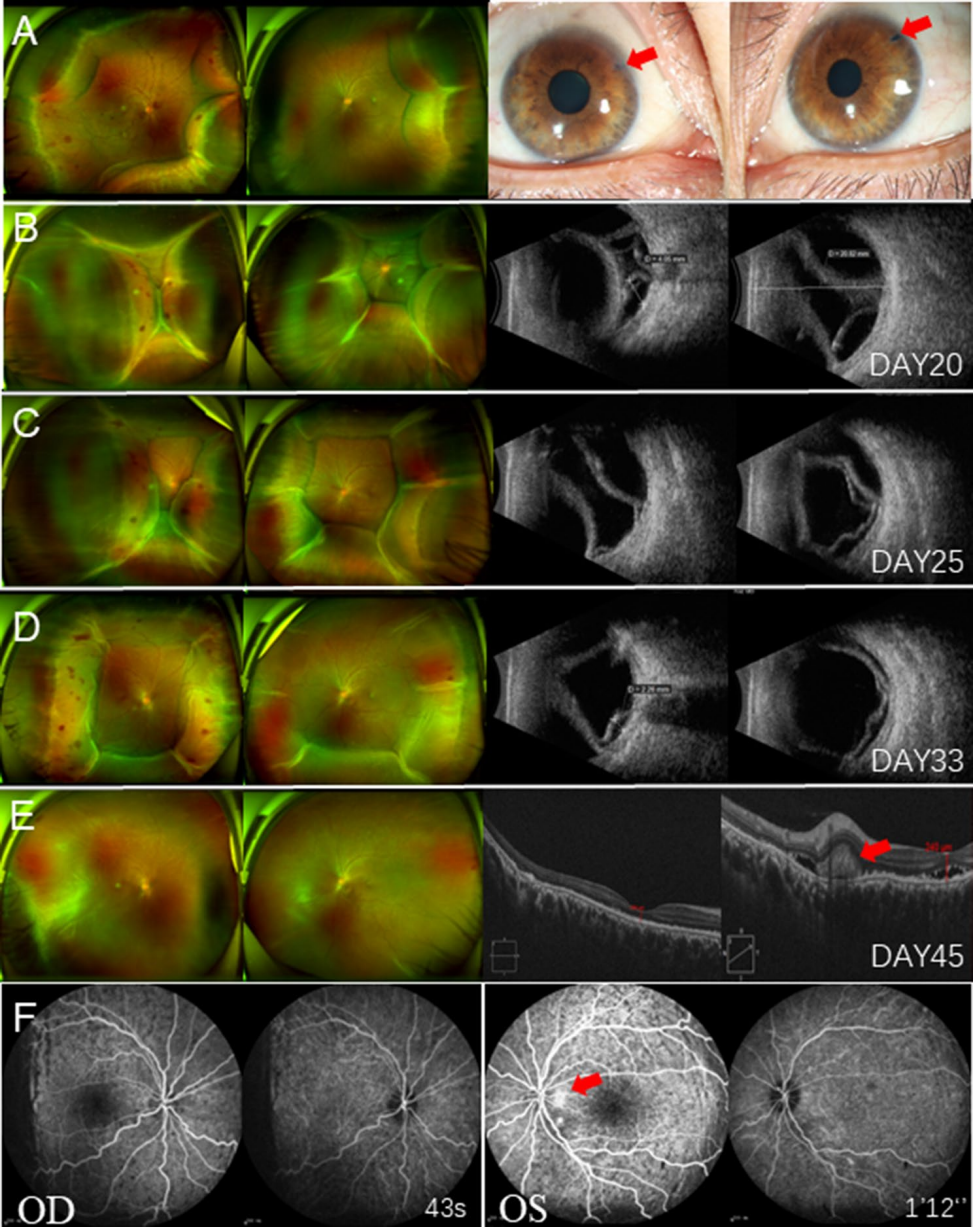
Bilateral fundus examination showed mound-like choroidal elevation with extremely shallow anterior chambers. Bilateral peripheral iridotomy was performed to reduce intraocular pressure (red arrowhead) (**A**). Twenty days later, choroidal detachment progressed rapidly, especially in the right eye, and bilateral periocular steroid injection was administered (**B**). The choroid gradually flattened from day 25 (**C**) to day 33 (**D**), with near-complete recovery by day 45(**E**). OCT of the left eye demonstrated a small amount of subretinal fluid (red arrowhead) (**E**). FFA and ICGA of both eyes showed leopard-spot pattern changes consistent with RPE alterations, with minimal fluorescein leakage noted in the temporal region of the left optic disc (red arrowhead) (**F**)

Systemic therapy with intravenous methylprednisolone, cyclophosphamide, hydroxychloroquine and telitacicept was initiated. However, ocular findings worsened. As shown in Fig. 1, Ultrasonography revealed massive 360-degree annular choroidal detachment (Fig. 1), and marked anterior displacement of the lens–iris diaphragm. Given the suspected inflammatory choroidal hyperpermeability, bilateral periocular corticosteroid injection of triamcinolone acetonide 20 mg, and then dexamethasone 5 mg were administered. The response was prompt. Choroidal detachment gradually resolved, the anterior chambers deepened(from 1.57 mm to 2.93 mm), and the retina reattached spontaneously. Fundus angiography revealed leopard spots in both eyes, with a small amount of exudate on the temporal side of the left optic disc. One month later, BCVA improved to 20/50 in both eyes, with normalised intraocular pressure. The patient reported significant improvement in vision and quality of life within two weeks after the periocular injection, and expressed gratitude for the rapid relief of ocular pain.

## Discussion

Classically, UES is attributed to impaired transscleral outflow and vortex vein congestion secondary to scleral thickening, most often in eyes with short axial length [2]. Our patient, however, had a normal axial length. Nanophthalmos was unlikely because axial length was within the normal range. Other differential diagnoses, including VKH disease, posterior scleritis, rhegmatogenous retinal detachment and drug-induced ciliochoroidal effusion, were considered but were not supported by the clinical history, B-scan/UBM findings, angiographic features and absence of retinal breaks or relevant drug exposure. Although idiopathic UES is classically regarded as a diagnosis of exclusion, type III UES has been associated with mechanisms beyond scleral thickening, including altered choroidal vascular permeability and non-specific choroidal inflammation [3]. In this context, active SLE may have acted as a systemic trigger producing a type III UES-like effusion pattern rather than a purely idiopathic form of UES. These findings suggest a secondary mechanism. We propose that severe hypoalbuminaemia and immune-mediated choroidal vascular hyperpermeability acted synergistically to promote excessive fluid accumulation within the suprachoroidal space. Fluorescein angiography was informative. Unlike lupus choroidopathy, which typically demonstrates diffuse multifocal pinpoint leakage, our patient showed only focal peripapillary leakage, arguing against classical diffuse inflammatory lupus choroidopathy as the sole dominant mechanism. A limitation of this case is that FFA and ICGA were not available at the earliest stage because angiography was not feasible during acute ocular and systemic deterioration.

Adjunctive periocular corticosteroid administration appeared to accelerate fluid resorption and anatomical recovery. Gazal Patnaik et al. [4] reported a case of a patient who showed a poor response to systemic corticosteroid therapy. Subsequently, posterior subtenon injections were administered. Visual improvement was then observed. Secondary angle-closure glaucoma in this context resulted from anterior displacement of the lens–iris diaphragm due to massive effusion. Similar mechanisms have been described in reports of SLE-associated choroidal effusion [5, 6]. These reports suggest choroidal effusion may be a part of polyserositis. Uveal effusion with secondary glaucoma may be a sign of systemic lupus erythematosus. Similarly, a 38-year-old female has been reported with Sjögren’s syndrome presenting initially as UES, the authors suggest that systemic factors may contribute to the pathogenesis of UES [7].

Systemic immunosuppression is essential for controlling active SLE, but ocular effusion may persist or progress during the early phase of systemic treatment. Periocular corticosteroid therapy may provide higher local ocular drug exposure, helping to suppress choroidal/ciliary vascular hyperpermeability and accelerate fluid resorption.

The role of PI in this setting should be interpreted with caution. PI was performed initially because the mechanism of acute bilateral angle closure was not yet clear and intraocular pressure was elevated. Subsequent ultrasonography and UBM demonstrated that angle closure was mainly caused by anterior displacement of the lens–iris diaphragm secondary to massive uveal/ciliochoroidal effusion, rather than pupillary block. Therefore, PI is unlikely to be definitive treatment in this mechanism, and treatment should primarily target the underlying effusion and systemic disease activity. A possible contribution from PI-related inflammation or intraocular pressure fluctuation cannot be excluded [8].

This case highlights the need to consider the patient’s overall systemic conditions, because ophthalmologic disease some times is the initial manifestation of systemic disease. In addition to systemic treatment, local ocular therapy is also crucial. Future studies are needed to investigate the impact of systemic factors on the pathogenesis of UES.

## Data Availability

The datasets used and/or analysed during the current study are available from the corresponding author on reasonable request.

## Abbreviations

ANA: Antinuclear Antibody
ANSA: Anti-nucleosome Antibody
ARPA: Anti-ribosomal P Protein Antibody
BCVA: Best-Corrected Visual Acuity
C3: Complement 3
C4: Complement 4
CRP: C-Reactive Protein
ESR: Erythrocyte Sedimentation Rate
SLE: Systemic Lupus Erythematosus
UES: Uveal Effusion Syndrome
PI: Peripheral iridotomy
